# The Prevalence of Early Implant Failure and Associated Risk Factors: A Retrospective Study

**DOI:** 10.4317/jced.63517

**Published:** 2026-02-26

**Authors:** U-rachanee Yuyuen, Kanyawat Rattanasuwan, Akkapol Banlue

**Affiliations:** 1 Department of Oral Medicine and Periodontology, Faculty of Dentistry, Mahidol University; 2 Maha Chakri Sirindhorn Dental Hospital, Faculty of Dentistry, Mahidol University

## Abstract

**Background:**

This retrospective study aimed to evaluate the prevalence and associated factors of early implant failure at Maha Chakri Sirindhorn Dental Hospital, Faculty of Dentistry of Mahidol University.

**Material and Methods:**

All implant placements performed between 2016 and 2023 were collected from the hospital's database (SSB program) using specific treatment codes. Failed implants were retrieved from the claimed implant list and categorized as early or late failures based on the timing of final restoration. Early failures were selected for further analysis, with data extracted from their respective treatment records, including patient's demographic data, periodontal status, and implant's characteristics.

**Results:**

A total of 7,368 dental implants were placed, of which 114 failed before final restoration and were subsequently removed. The prevalence of early implant failure was 1.55%. Univariate analysis revealed associations between early implant failure and a history of periodontitis, diameter of implant, location of implant placement, level of implant platform as determined by radiographic analysis, covering materials of implants. Moreover, length of implant was also having a trend toward association with early implant failure (p=0.052). From multivariate logistic regression analysis, only diameter (p=0.003) and length of implant (p=0.022) were associated with early implant failure.

**Conclusions:**

Our findings suggest that implant diameters less than 3.5 mm and lengths less than 10 mm are associated with an increased risk of early implant failure. Further research is warranted to clarify the influence of other potential contributing factors.

## Introduction

Tooth loss constitutes a significant global health concern. Consequently, planning tooth substitutes with patients is crucial. Contemporary dental practice has witnessed a surge in the popularity of dental implants, which are characterized by a high success rate of up to 98.7% (1). However, implant failure rates range from 1.5% to 4.8% (2 - 5) and they are categorized as early or late based on the timing of failure, typically defined by the time of loading or prosthesis placement (3 , 6 , 7). Numerous factors contribute to implant failure and can be divided into three subgroups: patient-related, tooth-related, and implant-related factors. Patient factors include age, gender, systemic diseases such as diabetes mellitus (DM) and osteoporosis, the utilization of antiresorptive medications, and smoking habits. Younger patients have been associated with early implant failure (5 , 8). While women tend to experience minimal bone loss in early implant failure (8), men also have an influence on early implant loss, with an odds ratio (OR) of 1.97 (1). DM, a chronic metabolic disease characterized by elevated blood glucose levels (9), can increase implant loss rates when poorly controlled (10). Nonetheless, well-controlled DM typically results in implant success rates comparable to healthy individuals (11). Osteoporosis may represent another potential contributing factor. While low-dose oral bisphosphonates generally do not impair implant treatment, high doses or prolonged use may increase the risk of medication-related osteonecrosis of the jaw (MRONJ) (12). Smoking exerts a deleterious effect on oral health, including both mucosa and the oral cavity. Smoking at the time of implant placement (first-stage surgery) has been strongly associated with early implant failure (13). Tooth factors also exert a significant influence on implant outcomes. An in vivo study (14) demonstrated a correlation between untreated experimental periodontitis and compromised osseointegration in implants with delayed placement. In contrast, no implant failures were observed in the non-periodontitis group. A history of periodontitis increases the risk of early mucosal inflammation, subsequent bone loss, and the development of peri-implantitis (15). A review article (6) found that two out of ten studies reported a higher risk of early implant loss in periodontally susceptible patients. Periodontitis-related bone loss may be a predictor of early implant loss. Finally, regarding implant factors, numerous studies have identified implant diameter and length as significant factors in early implant failure, with narrower and shorter implants demonstrating higher rates of loss (3 , 4 , 7 , 13). The effect of implant location remains inconclusive (1 , 5). A meta-analysis demonstrated significantly higher early implant failure rates in the maxilla, particularly in completely edentulous maxillae (13). Post-extraction alveolar ridge resorption can compromise implant stability, necessitating guided bone regeneration (GBR) procedures. However, these procedures may increase the risk of early implant failure (4). In the posterior maxilla, a sinus lift may be required due to maxillary sinus pneumatization. A high success rate for both grafts and implants has been observed when the residual bone height exceeds 4 mm (16). To mitigate the risk of early implant failure, antibiotic prophylaxis is often used either preoperatively or postoperatively at the time of implant placement (17). As mentioned above, several factors contribute to early implant failure, some of which remain unclear and necessitate further investigation. There is insufficient evidence and comprehensive data analysis pertaining to early implant failure and its associated factors within the Thai population. Therefore, this retrospective study aims to evaluate the prevalence and factors associated with early implant failure at Maha Chakri Sirindhorn Dental Hospital, Faculty of Dentistry, Mahidol University.

## Material and Methods

This retrospective study analyzed demographic and clinical data extracted from patient records at Maha Chakri Sirindhorn Dental Hospital. To ensure blinding, a designated individual, independent of the examiner, assigned a unique code to each patient chart, concealing patient identities, including names and hospital numbers. - Sample selection A Previous study reported the prevalence of early implant failure was 1.5% (2). With a 95% confidence interval (CI) of 2% ± 0.3%, a sample of 6,307 was needed (p=0.015, error = 0.003).

(1) $$n=\frac{z_{1-\frac{\alpha }{2}}^{2}p(1-p)}{d^{2}}$$

Patient data from 2016 to 2023 were collected from the hospital information system using specific treatment codes. Failed implants were identified from the claimed implant list and subsequently categorized as either early or late failures based on timing of the final restoration. Patients exhibiting early implant failures were selected for further analysis, including demographics, periodontal status, and implant characteristics. A nested case-control study design was performed, with cases defined as patients experiencing early implant failure and controls as patients without failure. Given the low prevalence of early implant failures, all cases were included. Controls were randomly selected with the case:control ratio of 1:2. In instances where a patient presented with multiple explanted implants, only one was randomly selected for inclusion in the analysis using a computerized randomization process. - Inclusion criteria Treatment records of patients who received implant placement between 2016 and 2023. Patients who had a history of at least one failed dental implant removal prior to final restoration. Patient medical and dental history, implants brand and design were available. - Exclusion criteria Implants that were explanted at the time of insertion due to no primary stability. Implants that were explanted after achieving osseointegration, but were deemed unrestorable due to improper positioning. - Data collection Patient factors included gender, age at the time of implant placement, DM, osteoporosis, antiresorptive drugs, and smoking status as shown in treatment records. DM was defined as HbA1C 6.5% or fasting plasma glucose (FPG) 126 mg/dL or 2-hour plasma glucose (PG) 200 mg/dL or random plasma glucose 200 mg/dL (9). Patients with HbA1C &lt;7% were considered to have controlled DM, and those with HbA1C 7% were considered to have uncontrolled DM (18). Osteoporosis was recorded from treatment records. Smoking status was categorized as a smoker and a non-smoker. Periodontal status, a tooth factor, was assessed. Patients with a history of periodontitis, defined as a PSR score of 3 or 4 or radiographic evidence of bone loss, were categorized as having treated or untreated periodontitis. Patients who received scaling and root planing were recorded as treated periodontitis. Implant factors included implant manufacturer (brand, type, design, diameter, length, and surface) implant location (anterior-posterior, maxilla-mandible, partial-complete edentulous), level of implant platform (crestal, sub-crestal) determined radiographically, implant covering materials (cover screw, healing abutment, temporary restoration), timing of implant placement (immediate, early, late), cause of tooth loss at the implant site, history of guided bone regeneration or sinus lift procedure at the implant area, type of materials used and the interval between these procedures and implant placement, antibiotics use at implant placement (no-antibiotics, pre-operative antibiotics, post-operative antibiotics, both pre- and post-operative antibiotics), number of removed implants per patient, interval from implant placement to removal, and experience of operators (specialist, resident in training). - Statistical Analysis Continuous and categorical variables were described using means and standard deviations, and counts and percentages, respectively. In the univariable analysis to determine the effect of each factor on early failure, the two-sample t-test and Mann-Whitney U test were used for quantitative variables with and without normal distribution respectively. Pearson's chi-square test and Fisher's exact test were applied to qualitative factors. Variables with a univariable p-value less than 0.10 were entered into multivariable analysis using multiple binary logistic regression as in previous study (19). Results were presented as adjusted OR with 95% CI and p-values. A p-value &lt; 0.05 was considered statistically significant. All statistical analyses were performed using IBM SPSS Version 28.

## Results

A total of 7,368 dental implants were placed at Maha Chakri Sirindhorn Dental Hospital between 2016 and 2023. Of these, 114 dental implants failed before the final restoration and were subsequently removed, resulting in an early implant failure prevalence of 1.55%. These 114 early implant failures affected a total of 96 patients, as some experienced multiple failures. All patients with implant failures were included in the analysis, with controls randomly selected at a 1:2 case-control ratio, resulting in 192 patients in the control group. As shown in Table 1, none of the patient factors showed a statistically significant difference.

Table 1

In the early implant failure group, 43 patients had a history of periodontitis, while 53 did not (Table 2).

Table 2

Of those with a history of periodontitis, 20 patients remained untreated, and 23 had received periodontitis treatment. Untreated periodontitis significantly increased the risk of early implant failure by 2.12-fold compared to those with no history of periodontal disease (p = 0.033; 95% CI, 1.07-4.24). Treated periodontitis was not associated with an increased risk of early implant failure compared to no history of periodontal diseases (p = 0.909). Regarding implant characteristics in the early implant failure group, several brands were used. The main reasons for tooth loss at the implant site were tooth fracture (34.7%), caries (28.6%), and periodontitis (12.2%). Univariate analysis of the association between implant characteristics and early implant failure revealed that diameter, location, the level of implant platform (from radiographs), and implant covering materials were significantly associated with early implant failure (Table 3).

Table 3

Length also demonstrated a trend toward an association (p=0.052). DM, history of periodontitis, implant diameter, implant length, implant location, implant platform level (from radiographs), implant covering materials, and implant placement timing were evaluated in a multiple logistic regression model (Table 4).

Table 4

A significant association with increased risk of early implant failure was observed for implant diameter and implant length. An implant diameter of less than 3.5 mm was associated with a sixfold increase in the risk of early implant failure (OR, 6.21; 95% CI, 1.88-20.55; p=0.003). Similarly, an implant length of less than 10 mm was associated with a twofold increase in the risk of early implant failure (OR, 2.06; 95% CI, 1.12-3.82; p=0.022).

## Discussion

The prevalence of early implant failure in this study was 1.55%. Univariate analysis identified a history of periodontitis, implant diameter, implant location, implant platform level (from radiographs), and implant covering materials as potential risk factors. Implant length also trends toward an association. However, only implant diameter and length were significantly associated with early implant failure in the multiple logistic regression model. This prevalence is comparable to the 1.5% reported by Friberg et al. (2). This similarity may be attributed to the fact that the operators in our study were primarily specialists in oral and maxillofacial surgery or implantology with extensive experience. Patients with untreated periodontitis had a 2.12-fold increased risk of early implant failure compared to healthy patients, corroborating the findings of a previous study by Olmedo-Gaya et al. (20). Their research indicated a significantly higher risk of failure in patients with severe periodontal disease, characterized by attachment loss exceeding 3 mm at 67-100% of sites. Jansson et al. (21) employed PCR to detect periodontal pathogens and discovered that Prophyromonas gingivalis was the most common microorganism detected around both teeth and implants. Similarly to Korsch et al. (22), Fusobacterium nucleatum and Porphyromonas gingivalis were abundant in cases of both early and late implant loss. The remaining teeth in periodontitis patients may serve as a reservoir for bacterial colonization at the implant site. Other studies (15), including the AAP/EFP 2018 World Workshop on Periodontology (23), have identified a history of periodontitis as a risk factor for peri-implantitis. Although untreated periodontitis was associated with early implant failure in the univariate analysis, this association was not maintained in the multivariate analysis. DM has been studied in relation to the risk of both implant failure and peri-implantitis (10 , 11 , 23). A systematic review and meta-analysis revealed that poorly controlled DM is associated with a 77.7% higher risk of implant loss compared to healthy patients (10). However, our study did not demonstrate this association. Although there was a trend between DM and early implant failure (p=0.081), it did not reach statistical significance. This discrepancy may be due to good glycemic control in some patients, potentially explaining why the risk was not as high as previously reported. Well-controlled diabetic patients have similar implant placement success rates to healthy individuals (11). Unfortunately, HbA1C data was unavailable, so we could not determine whether the patients had controlled DM. Currently, numerous studies indicate that vitamin D deficiency may adversely affect implant integration and contribute to implant failure. Kwiatek et al. (24) reported that elevated vitamin D levels on the day of surgery were correlated with enhanced peri-implant bone growth at 6 and 12 weeks. Additionally, Fretwurst et al. (25) documented clinical case reports linking early implant failure to vitamin D insufficiency, suggesting that vitamin D supplementation may improve the success of implant replacement. Vitamin D is necessary for calcium absorption, and its deficiency can lead to osteoporosis and an increased risk of bone fractures. Unfortunately, the number of osteoporosis cases in our study was insufficient for meaningful analysis. Furthermore, due to the limitations of the retrospective study, vitamin D levels were not documented in patient charts, thereby precluding inclusion of this factor in this study. A previous study indicated that smoking was a significant predictor of early implant failure, with an OR of 1.7 (95% CI, 1.3-2.3) (13). Our study did not confirm this association due to insufficient information in the treatment records. Patients were not explicitly classified as current or former smokers, and the data relied on self-reporting. Consequently, patients who did not report smoking on the day of history-taking may have been lifelong non-smokers or may have quit previously. Multiple logistic regression identified an implant diameter of less than 3.5 mm as a significant predictor of early implant failure, consistent with previous research (3 , 7). This finding may be attributed to the limited surface area contact of narrow implants, which are often used in cases of insufficient buccolingual alveolar ridge width or limited mesiodistal space. The placement of wider implants may be more effective in dissipating the forces and thereby reducing the stress on the bone surrounding the implant (26). Implant length of less than 10 mm also emerged as a significant risk factor in this study, which is consistent with the other studies (3 , 4 , 13 , 20). This may be due to a reduced bone contact surface area, as shorter implants are often placed in areas with inadequate bone quality. Short implants are more likely than standard implants to cause higher strain in the cancellous bone and greater stress levels in the peri-implant bone (27). There might be a higher chance of implant failure as a consequence. However, they may be considered an alternative when implant placement is close to vital structures, for example, the maxillary sinus or inferior alveolar nerve, necessitates a less invasive approach. Feldman et al. (28) showed that short implants (10 mm or less) placed in poor bone quality had lower cumulative survival rates compared to standard-length implants. Conversely, some studies have suggested a trend toward increased failure rates for short implants, although these findings were not statistically significant (7 , 19). Using short implants with wider diameter has been found to generate lower stress than longer implants with narrow diameters (26). The anterior mandible was associated with early implant failure in the univariable analysis compared to the posterior mandible, but this association was not observed in the multiple logistic regression analysis. This could be due to the dense cortical bone in the anterior mandible, which may lead to increased bone heat during implant drilling and has a lower number of blood vessels. Lin et al. (1) also reported that implants placed in the anterior mandible were associated with early implant loss. Conversely, previous studies have linked the mandibular posterior region and completely edentulous maxilla to a higher risk of early implant failure (2 , 5). In the present study, these differences were not statistically significant due to the small number of completely edentulous patients. Univariate analysis revealed a significant association between cover screw utilization and early implant failure compared to healing abutments. This could be explained by cover screws being typically used in cases of primary instability or bone augmentations, where there was initially insufficient bone. Univariate analysis also indicated a correlation between the crestal level of the implant platform and early implant failure. Although no studies have directly addressed the relationship between platform level and early implant failure, research has explored marginal bone loss around implants. A systematic review suggested that implants placed in the subcrestal position had less marginal bone loss change compared to those in the crestal position (29). Nonetheless, other studies have suggested that both crestal and subcrestal levels are acceptable and can maintain stability around dental implants (30). Early implant placement tended to be associated with a higher risk of early implant failure compared to immediate placement. However, this difference was not statistically significant in the multivariate analysis. This finding agrees with previous studies which reported no difference in implant outcomes (31 , 32). This study, conducted at one of Thailand's largest hospitals in Nakhon Pathom, represents the first assessment of early implant failure prevalence and associated factors. Data were collected from 7,368 dental implants placed between 2016 and 2023. Implant numbers were used as patient identifiers. To minimize bias, the control group selection was randomized to match the year of the failure group. This retrospective study has some limitations. The small sample size limited the statistical power for some factors. Data limitations included missing HbA1C records in diabetic patients and incomplete smoking histories (quantity and duration). Incomplete treatment records and illegible handwriting also presented challenges. Furthermore, the absence of radiographs in some cases and deviations from the parallel technique precluded accurate assessment of implant platform levels. To enhance statistical power, we recommend extending the data collection period. HbA1C records and cigarette consumption should be included in patient charts, and all clinical practices should adhere to standard guidelines. Further research is necessary to investigate the specific relationship between early implant failure, HbA1C levels, and smoking habits. In the clinic, we advocate for an optimal treatment plan for implant placement, such as GBR, to achieve precise lengths and diameters, hence minimizing the chance of early implant failure.

## Conclusions

In conclusion, the prevalence of early implant failure in this study was 1.55%. Within the limitations of this study, our findings suggest that implant diameters less than 3.5 mm and lengths less than 10 mm are associated with an increased risk of early implant failure. Further research is needed to elucidate the association of early implant failure with other factors.

## Figures and Tables

**Table 1:** Association between patient’s factors and failure using univariable analysis.

	Number (%)			
Factors	Survived implants (n=192)	Failed implants (n=96)	OR (95% CI)	p-value
Gender Male Female	80 (41.7)112 (58.3)	45 (46.9)51 (53.1)	10.81 (0.49, 1.33)	0.401+
Age, years: mean ± SD Age range (years) Mean ± SD	19-8457.7 ± 10.5	17-7857.4 ± 14.0	-	0.829++
Diabetes Mellitus* Yes No	20 (10.4)170 (89.6)	17 (17.7)79 (82.3)	1.85 (0.92, 3.72)1	0.081+
Osteoporosis Yes No	1 (0.5)191 (99.5)	1 (1.0)95 (99.0)	-	-
Antiresorptive drugs† Yes No	0191 (100.0)	2 (2.1)94 (97.9)	-	-
Smoking‡ Yes No	12 (6.6)170 (93.4)	11 (12.1)80 (87.9)	1.95 (0.82, 4.60)1	0.123+

+ Pearson’s chi-square test, ++ Mann-Whitney U testAbbreviation: SD, standard deviation; OR, Odds ratio; CI, confidence interval.N/A information: * 2 cases in survived, † 1 case in survived, ‡ 10 cases in survived and 5 cases in failed.

**Table 2:** Association between periodontal status and failure using univariable analysis.

	Number (%)			
Factors	Survived implants (n=192)	Failed implants (n=96)	OR (95% CI)	p-value
History of periodontitis No Yes, Treated Yes, Untreated	118 (61.5)53 (27.6)21 (10.9)	53 (55.2)23 (24.0)20 (20.8)	10.97 (0.54, 1.74)2.12 (1.07, 4.24)	0.9090.033

Pearson’s chi-square test.

**Table 3:** Association between implant’s characteristics and failure using univariable analysis.

Factors	Number (%)		OR (95% CI)	p-value
	Survived (n=192)	Failed (n=96)		
Diameter (mm) <3.5 ≥3.5	5 (2.6)187 (97.4)	14 (14.6)82 (85.4)	6.39 (2.23, 18.31)1	<0.001
Length (mm) <10 ≥10	47 (24.5)145 (75.5)	34 (35.4)62 (64.6)	1.69 (0.99, 2.88)1	0.052
Location Anterior maxilla Anterior mandible Posterior maxilla Posterior mandible	24 (12.5)4 (2.0)60 (31.3)104 (54.2)	20 (20.8)8 (8.4)24 (25.0)44 (45.8)	2.04 (1.10, 4.11)4.31 (1.23, 15.11)0.94 (0.52, 1.71)1	0.0460.0220.849
Level of implant platform† Sub-crestal Crestal	141 (73.8)50 (26.2)	57 (61.3)36 (38.7)	11.78 (1.05, 3.02)	0.031
Covering materials of implants‡ Healing abutment Cover screw Temporary restoration	153 (79.7)38 (19.8)1 (0.5)	64 (67.4)31 (32.6)0	11.95 (1.12, 3.40) a-	0.018a
Timing of implant placement§ Immediate placement Early placement Late placement	27 (14.3)39 (20.6)123 (65.1)	7 (7.3)24 (25.0)65 (67.7)	12.39 (0.89, 6.44)2.07 (0.84, 5.09)	0.0850.115

Pearson’s chi-square test.a Compare cover screw and healing abutmentAbbreviation: GBR, Guided Bone RegenerationN/A information: † 1 case in survived and 3 cases in failed, ‡ 1 case in failed, § 3 cases in survived

**Table 4:** Relationship between factors and early implant failure: multiple logistic regression.

Factors	Adjusted OR	95% CI for OR	p-value
Diabetes Mellitus No Yes	11.95	(0.89, 4.26)	0.094
History of periodontitis No Yes, Treated Yes, Untreated	10.971.81	(0.52, 1.82)(0.84, 3.90)	0.9200.132
Diameter (mm) ≥3.5 <3.5	16.21	(1.88, 20.55)	0.003
Length (mm) ≥10 <10	12.06	(1.12, 3.82)	0.022
Location Posterior mandible Anterior maxilla Anterior mandible Posterior maxilla	11.452.130.75	(0.65, 3.27)(0.50, 9.09)(0.39, 1.43)	0.3650.3080.377
Level of implant platform from x-ray Sub-crestal Crestal	11.67	(0.95, 2.94)	0.075
Covering materials of implants Healing abutment Cover screw	11.08	(0.55, 2.11)	0.825
Timing of implant placement Immediate placement Early placement Late placement	12.251.60	(0.78, 6.55)(0.61, 4.21)	0.1360.343

4

## Data Availability

The datasets used and/or analyzed during the current study are available from the corresponding author.
